# IgG4-related prostatitis manifesting as urinary obstruction in a 28-year-old male

**DOI:** 10.1186/s12894-022-00980-2

**Published:** 2022-03-11

**Authors:** Aria Jazdarehee, Azin Ahrari, Drew Bowie, Silvia D. Chang, Henry Tran, Shahin Jamal, Luke Y. C. Chen, Karen C. Tran

**Affiliations:** 1 Faculty of Medicine, University of British Columbia, Vancouver, BC Canada; 2 Division of Rheumatology, University of British Columbia, Vancouver, BC Canada; 3 Department of Radiology, Vancouver General Hospital, University of British Columbia, 899 West 12th Avenue, Vancouver, BC V5Z 1M9 Canada; 4 Division of Urology, University of British Columbia, Vancouver, BC Canada; 5 Division of Hematology, University of British Columbia, Vancouver, BC Canada; 6 Division of General Internal Medicine, University of British Columbia, Vancouver, BC Canada; 7 Vancouver General Hospital, 7th Floor, 2775 Laurel Street, Vancouver, BC V5Z 1M9 Canada

**Keywords:** IgG4-related disease, Prostatitis, Sialadenitis, Priapism, Case report

## Abstract

**Background:**

Immunoglobulin G4-related disease (IgG4-RD) is a systemic lymphoproliferative disorder characterized by elevated serum IgG4 levels and tumefactive lesions that can involve nearly every organ system. Involvement of the prostate is rare but has been reported in limited cases.

**Case presentation:**

A 28-year-old man of Asian descent with a history of sinusitis and priapism presented to hospital with rigors and voiding symptoms. He was diagnosed with IgG4-RD one month prior to presentation, following pathological analysis of a submandibular mass that demonstrated chronic sclerosing sialadenitis. On presentation, white blood cell count, C-reactive protein, and prostate serum antigen levels were all within normal limits. Examination was notable for a large, firm prostate, and a foley catheter was inserted. Contrast CT of the abdomen was unremarkable. Further workup revealed elevated serum IgG4 levels (9.22 g/L) and he was subsequently started on prednisone 35 mg daily. Imaging to screen for systemic IgG4-RD involvement demonstrated paravertebral soft tissue involvement and he was given rituximab 1000 mg IV × 2 doses. MRI revealed diffuse prostatitis. Five days after starting prednisone and one day after his first dose of rituximab, he successfully passed trial of void and was discharged home.

**Conclusions:**

IgG4-related prostatitis is a rare and underrecognized manifestation of IgG4-RD. Our case highlights the need to consider IgG4-related prostatitis as an etiology of urinary obstruction in young individuals. Resolution of symptoms following treatment with steroids may be diagnostic of IgG4-related prostatitis, and may potentially avoid the need for invasive diagnostic procedures such as prostate biopsy.

## Background

Immunoglobulin G4-related disease (IgG4-RD) is an immune-mediated lymphoproliferative disorder characterized by elevated serum IgG4 levels and tumefactive lesions that can involve nearly every organ system [1, 2]. Histopathology is imperative to diagnosis and key features include lymphoplasmacytic infiltrates, storiform fibrosis, and IgG4 plasma cells [3]. These pathological characteristics are common across organs affected by the disease, which primarily include the pancreas, kidneys, orbital adnexa, and salivary glands [4].

In 2019, the American College of Rheumatology (ACR) and European League Against Rheumatism (EULAR) proposed a three-step classification system for the diagnosis of IgG4-RD [5]. A case meets criteria for IgG4-RD if: (1) At least one of 11 possible organs commonly affected by IgG4-RD are involved, (2) No specified exclusion criteria are met, and (3) Inclusion criteria are met above a specific scoring threshold. The 2019 ACR/EULAR criteria for IgG4-RD are outlined in Table 1.

**Table 1:** The 2019 American College of Rheumatology (ACR)/European League Against Rheumatism (EULAR) criteria for immunoglobulin G4-related disease (IgG4-RD) ( adapted from Wallace et al. 2019)

*Step 1: Must be clinical or radiological involvement of:*		
Pancreas Salivary glands Bile ducts Orbits	Kidney Lung Aorta	Retroperitoneum Pachymeninges Thyroid Gland
OR pathological evidence of an inflammatory process accompanied by a lymphoplasmacytic infiltrate in one of these same organs		
*Step 2: The following exclusion criteria must be applied:*		
*Clinical* Fever No response to glucocorticoids *Serological* Leukopenia and thrombocytopenia Peripheral eosinophilia Positive ANCA antibodies Positive SSA/Ro or SSB/La antibody Positive dsDNA, RNP or Sm antibody Other disease-specific autoantibody Cryoglobulinemia	*Radiological* Findings suspicious for malignancy or infection not fully investigated Rapid radiological progression Long bone abnormalities Splenomegaly *Known diagnosis of:* Multicentric Castleman’s disease Crohn’s disease or ulcerative colitis Hashimoto thyroiditis (if only thyroid affected)	*Pathological* Cellular infiltrates suggesting malignancy not fully investigated Markers consistent with inflammatory myofibroblastic tumor Prominent neutrophilic inflammation Necrotizing vasculitis Prominent necrosis Primary granulomatous inflammation Features of macrophage disorder
*Step 3: Inclusion criteria; total points must equal or exceed 20 to be classified as IgG4-RD*		
*Histopathology* Uninformative biopsy: 0 Dense lymphocytic infiltrate: + 4 Dense lymphocytic infiltrate and obliterative phlebitis: + 6 Dense lymphocytic infiltrate and storiform fibrosis: + 13 *Chest* None apply: 0 Peribronchovascular and septal thickening: + 4 Paravertebral band-like soft tissue in thorax: + 10 *Pancreas and Biliary Tree* None apply: 0 Diffuse pancreatic enlargement (loss of lobulations): + 8 Diffuse pancreatic enlargement + capsule-like rim with decreased enhancement: + 11 Pancreatic and biliary tree involvement: + 19	*Immunostaining* IgG4/IgG ratio 0–40% and number of IgG4 cells/hpf is 0–9: 0 IgG4/IgG ratio ≥ 41% and number of IgG4 cells/hpf is 0–9 OR ratio is 0–40% and cells/hpf ≥ 10: + 7 IgG4/IgG ratio 41–70% and number of IgG4 cells/hpf is ≥ 10 OR ratio is ≥ 71% and cells/hpf 10–50: + 14 IgG4/IgG ratio ≥ 71% and number of IgG4 cells/hpf is ≥ 51: + 16 *Serum IgG4 concentration* Normal or not detected: 0 > Normal to < 2 × ULN: + 4 2–5 × ULN: + 6 ≥ 5 × ULN: + 11	*Bilateral lacrimal, parotid, sublingual, and submandibular glands* None involved: 0 1 involved: + 6 2 + involved: + 14 *Retroperitoneum* None apply: 0 Diffuse thickening of the abdominal aortic wall: + 4 Circumferential or anterolateral soft tissue around the infrarenal aorta or iliac arteries: + 8 *Kidney* None apply: 0 Hypocomplementemia: + 6 Renal pelvis thickening/soft tissue: + 8 Bilateral renal cortex low-density areas: + 10

First, clinical or radiological involvement of one of 11 typical organs implicated with IgG4-RD must be established. Second, exclusion criteria consisting of 32 clinical, serological, radiological, and pathological items must be applied. Third, eight weighted inclusion criteria domains are applied- if a threshold score of 20 points is achieved, the case may be classified as IgG4-RD

Although IgG4-RD may affect nearly every organ system, genitourinary involvement is less commonly reported and typically manifests as retroperitoneal fibrosis [6]. Involvement of the prostate is less common, but IgG4-related prostatitis has been reported in limited case reports [7–15]. In the majority of described cases, those with prostatic involvement are elderly males over the age of 55 whose symptoms were initially attributed to benign prostatic hyperplasia (BPH) [8, 10]. Here we describe a case report of IgG4-related prostatitis in a 28-year-old male presenting with voiding symptoms and dysuria.

## Case presentation

**Fig. 1:**
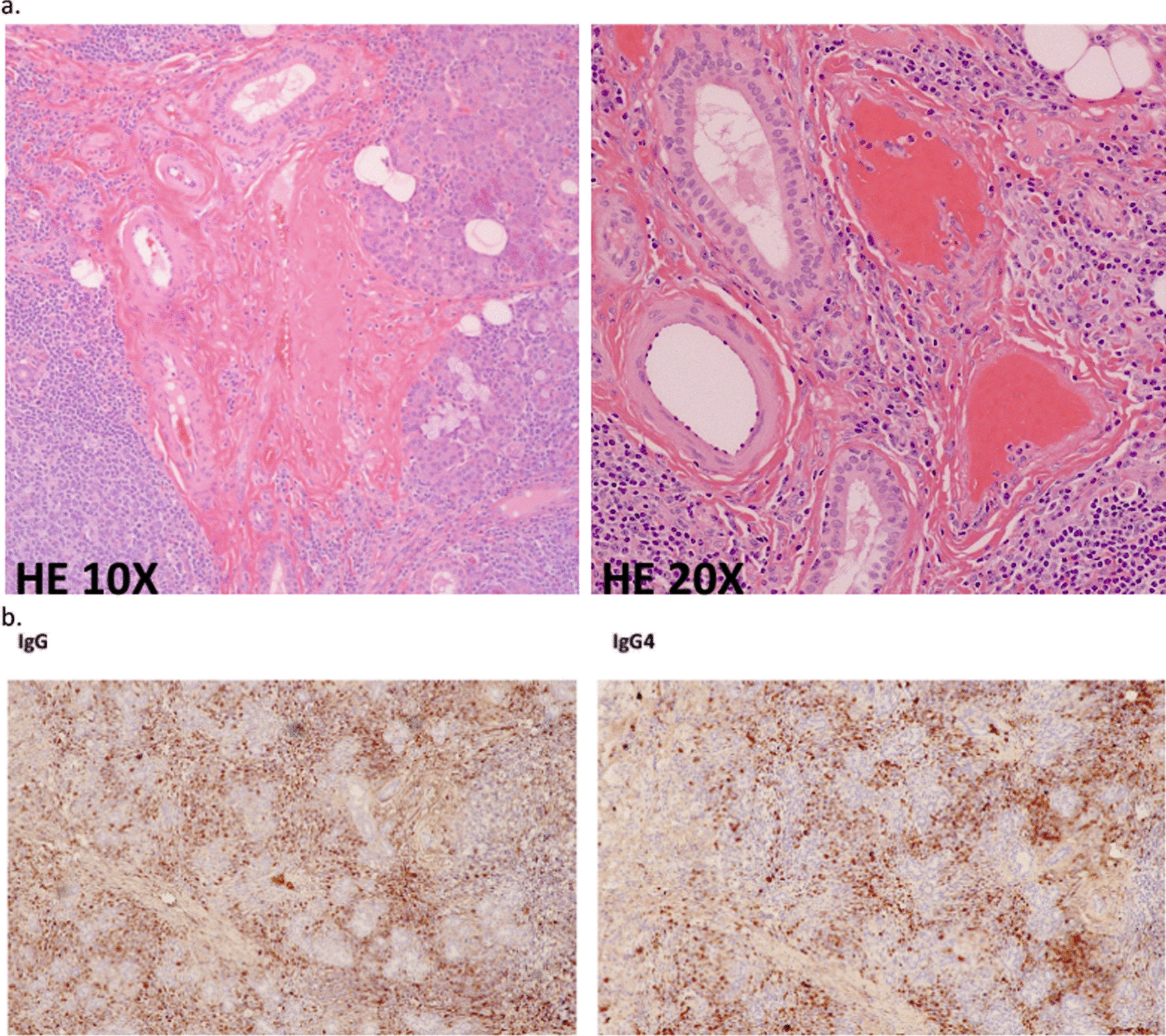
Pathological analysis of resected submandibular mass. **a** Analysis of the nodule demonstrating chronic sclerosing sialadenitis. **b** Immunohistochemistry staining revealing infiltration of IgG and IgG4 plasma cells

A previously healthy, 28-year-old man of Asian ethnicity presented to hospital with rigors and worsening voiding symptoms, including hesitancy, straining, and slow stream. One month prior to his presentation, he underwent surgical excision of a left-sided submandibular mass noted 1 year earlier. Initial biopsy of the left-sided submandibular mass revealed a 2.4 cm nodule with pathological features of reactive follicular lymphoid hyperplasia and no evidence of lymphoma. Resection of the mass was complicated by a postsurgical abscess two weeks later for which he required admission to hospital for drainage and antibiotic therapy with IV ceftriaxone and metronidazole. Pathological analysis of the nodule demonstrated chronic sclerosing sialadenitis with many areas showing greater than 100 IgG4-positive plasma cells per high-power field (Fig. 1). Analysis of associated lymph nodes demonstrated follicular hyperplasia and increased IgG4-positive plasma cells, particularly in the follicles.

The patient first developed urinary frequency and dribbling associated with suprapubic pain and dysuria two days after discharge from hospital for his postsurgical abscess. The following day, he developed rigors, but no fever, night sweats, hematuria, urinary incontinence, or back pain. Review of systems was significant for two-day history of constipation. Several days later, the patient was seen by his family doctor and started on ciprofloxacin 500 mg for 5 days and tamsulosin 0.8 mg daily. With no improvement in clinical symptoms and worsening rigors the following day, he presented to the hospital.

His past medical history was notable for a one-year history of sinusitis for which the patient received weekly immunotherapy, as well as a three-year history of intermittent priapism for which urology assessment was pending. The patient took no medications and had no known drug allergies.

On presentation to the emergency department, he was clinically stable with blood pressure 122/64, heart rate 83 beats per minute, and afebrile at 36.7° C. Digital rectal examination was unremarkable. Prostate exam noted a large and firm prostate. A bladder scan demonstrated a volume of 573 mL and a foley catheter was inserted. Laboratory investigations were significant for white blood cell count of 5.7 × 10^9^/L, serum creatinine of 76 µmol/L, and C-reactive protein of 1.7 mg/L. Blood cultures, urine culture and urinalysis were negative.

A contrast computed tomography (CT) scan of the abdomen demonstrated no stones or hydronephrosis, but a minimally enlarged prostate at 31 mm was noted. There was no evidence of retroperitoneal fibrosis. Subsequent tests for HIV, syphilis, gonorrhea, and chlamydia were negative, and prostate serum antigen (PSA) was normal at 0.77 µg/L. The patient was admitted to internal medicine for further workup of his urinary symptoms.

During his admission, he was assessed by rheumatology and hematology. Additional investigations revealed an elevated serum IgG levels of 18.6 g/L (6.7–15.2 g/L) as well as elevated serum IgG4 level at 9.22 g/L (0.052–1.250 g/L). Other IgG subclasses were within normal limits. Serum protein electrophoresis (SPEP) demonstrated an uneven distribution of polyclonal immunoglobulins towards the fast gamma region, a finding consistent with elevated serum IgG4 levels. Autoantibody screen was negative and complement levels were normal. Given the pathology of his submandibular mass was positive for IgG4, he was started on treatment for IgG4-RD with prednisone 0.5 mg/kg/day (35 mg daily) on day 2 of admission.

**Fig. 2:**
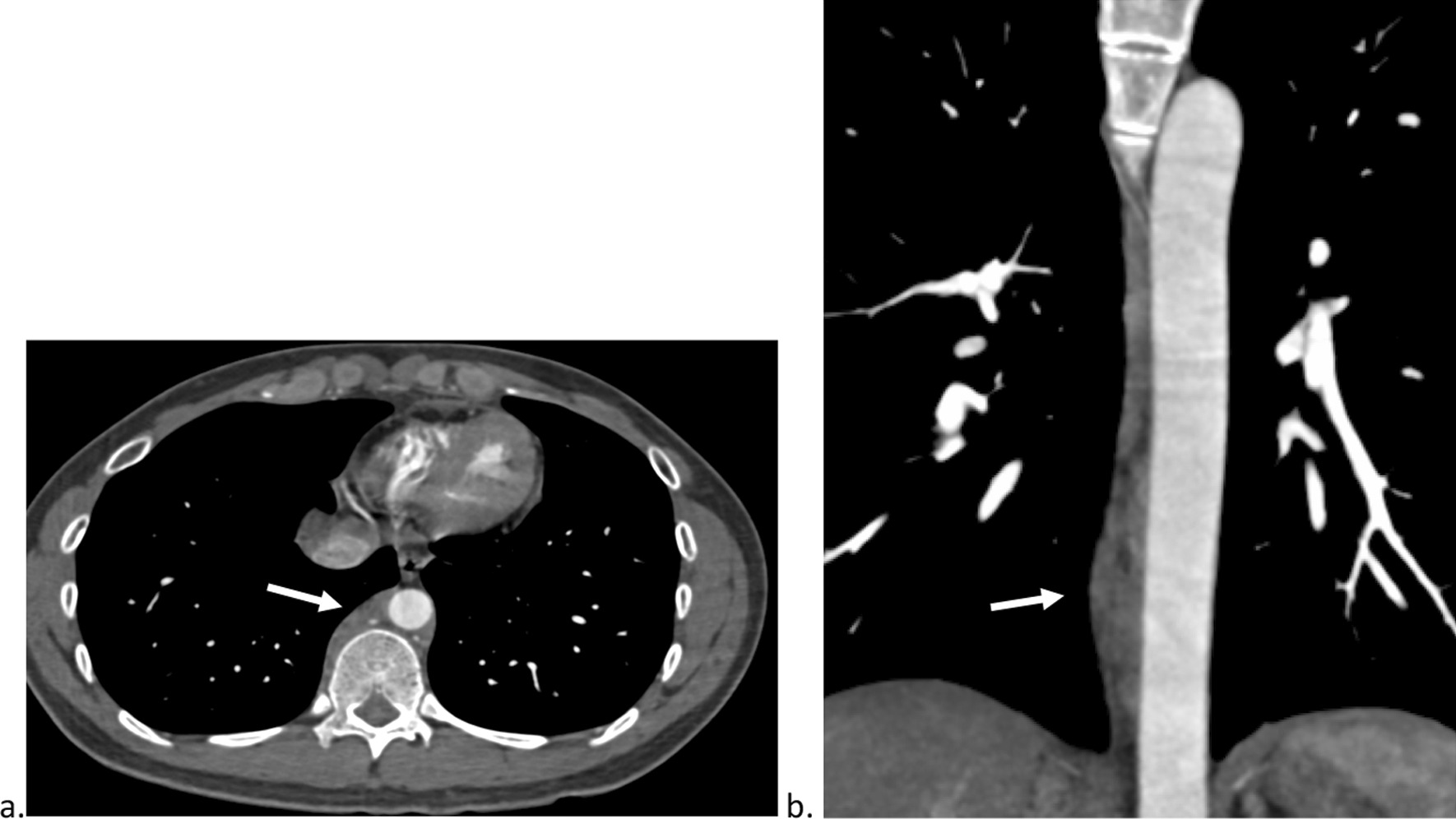
Axial (**a**) and coronal (**b**) images from a contrast-enhanced CT of the chest. Soft tissue density adjacent to the aorta and vertebral body (arrow) is evident

A CT chest was completed to evaluate for systemic IgG4 that demonstrated paraaortic and paravertebral soft tissue involvement at the levels of T8-12 (Fig. 2). Multisystem IgG4-RD involvement was suspected and therefore he was initiated on rituximab 1000 mg IV on day 6 of admission.

**Fig. 3:**
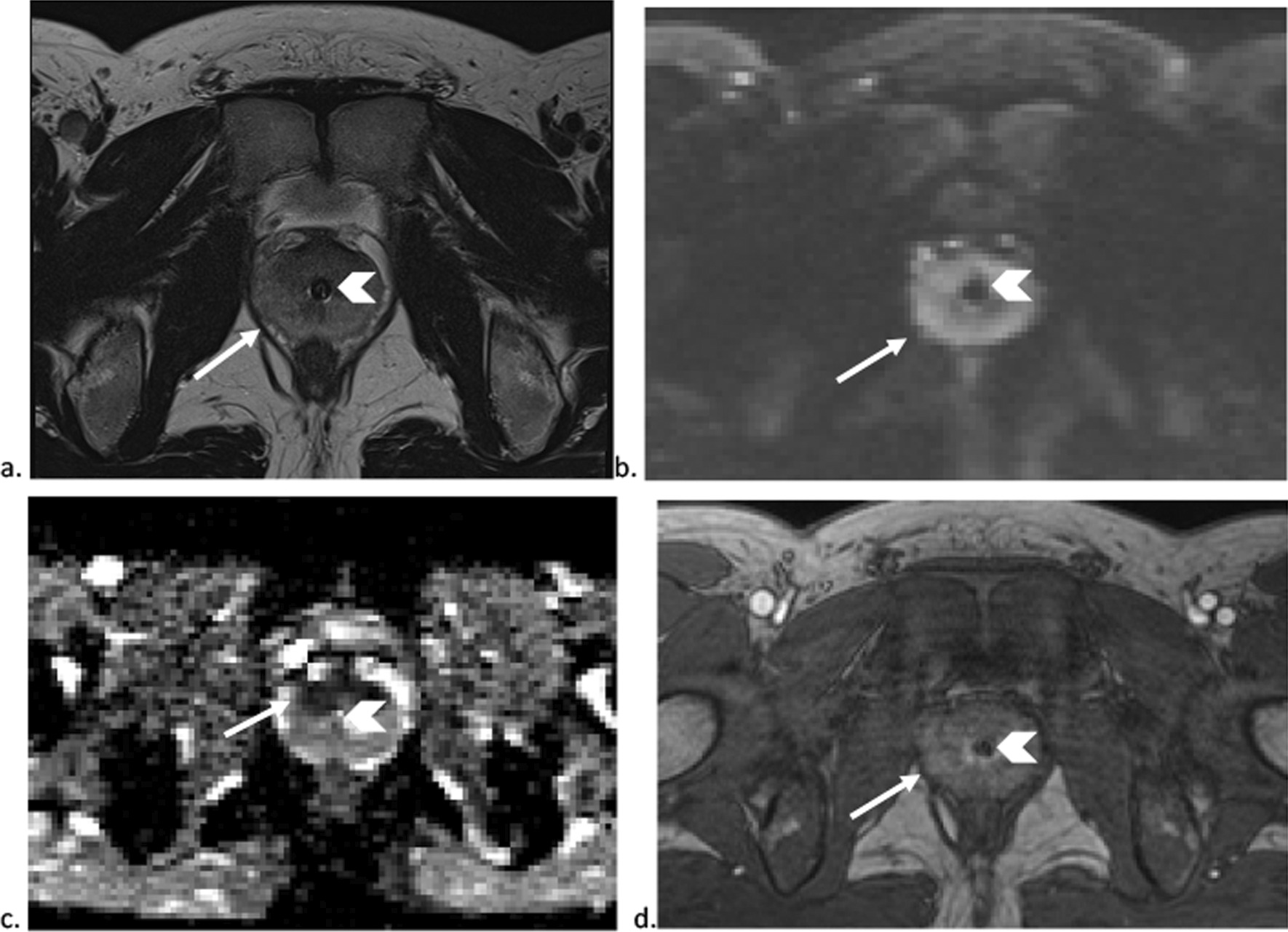
MRI of the prostate gland showing diffuse prostatitis. T2 weighted (**a**), high b value 1500 DWI (**b**), ADC map (**c**) and dynamic contrast-enhanced (**d**) images show the prostate gland with a volume of 30 cc with a Foley catheter in situ (arrowhead). The peripheral zone is diffusely mildly T2 hypointense (**a**) (arrow). There is diffuse restricted diffusion (**b**, **c**), which is greater in the transition zone with lower signal intensity on the ADC map (**c**) (arrow). There is diffuse mild (**d**) hypervascularity (arrow)

With respect to the patient’s urinary symptoms, he failed a trial of void day 2 of admission and subsequently a foley catheter was re-inserted. Urology was consulted to assess this patient. The prostate was mildly enlarged on magnetic resonance imaging (MRI), measuring 4.1 × 3.4 × 4.2 cm (30 cc), with evidence of diffuse prostatitis (Fig. 3). A cystoscopy was requested to rule out obstructive causes, but was not completed in hospital. Given the patient’s young age and low PSA levels, it was presumed that his enlarged prostate represented IgG4-RD involvement rather than acute bacterial prostatitis, BPH, or malignancy. Five days after starting prednisone and one day after receiving rituximab, he successfully passed trial of void and was discharged home, with outpatient follow up with rheumatology, hematology, and urology.

With prednisone 35 mg daily and rituximab, he did not have recurrence of urinary symptoms. Furthermore, there was no recurrence of submandibular mass. Interestingly, he was previously experiencing sinusitis symptoms treated under supervision of ENT that completely resolved with prednisone. Rituximab 1000 mg IV was given 41 days after initial dose to complete induction. Prednisone was gradually tapered over seven weeks.

In addition to clinical improvement, biochemical improvement with reduction of serum IgG4 levels was also noted. Given his clinical remission without evidence of recurrence, no further treatment for IgG4-RD was offered. He continues to be monitored clinically and with serial serum IgG4 levels. He remains in remission four months after discontinuing prednisone and receiving rituximab.

**Fig. 4:**
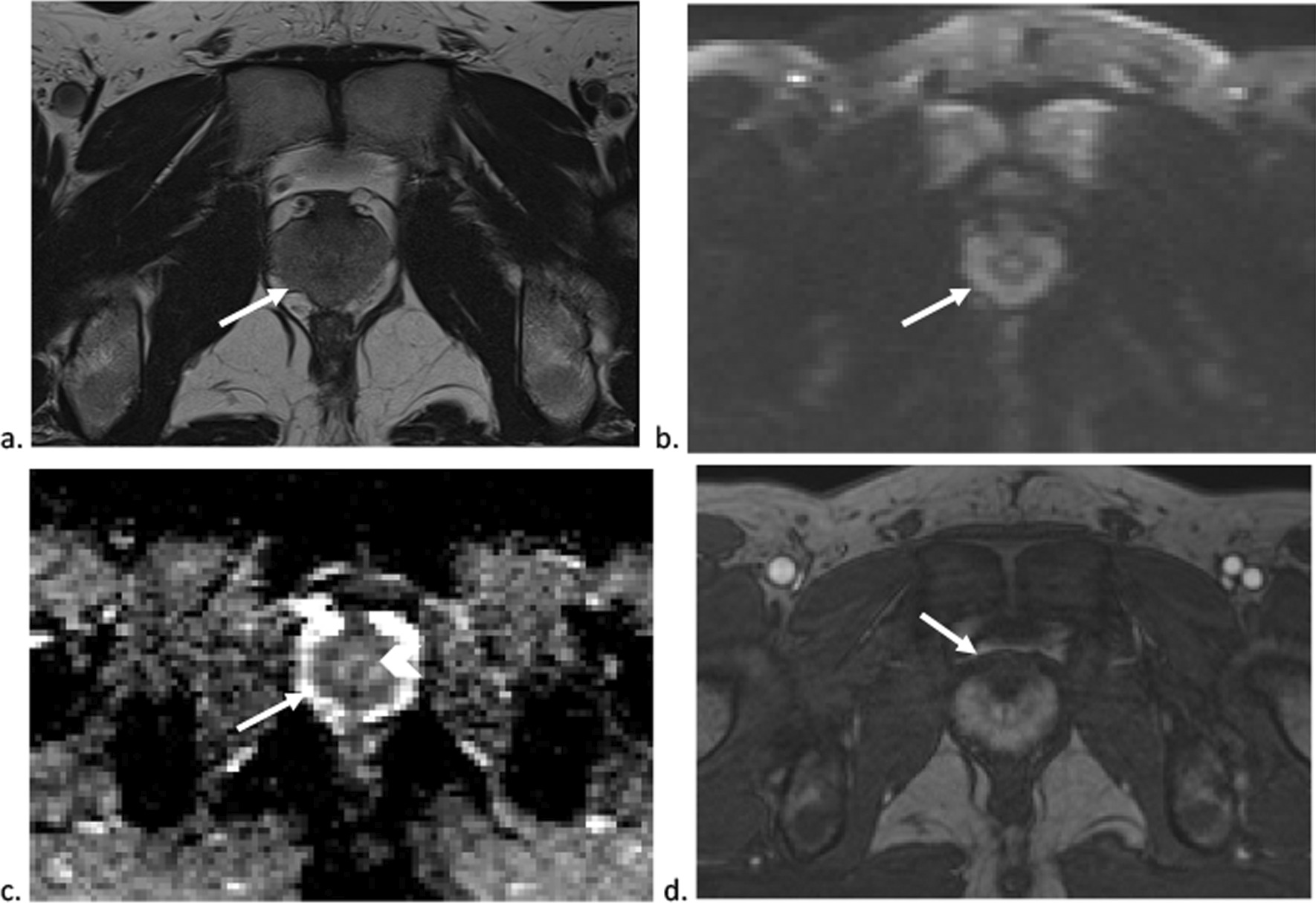
Follow-up MRI of the prostate gland four months later. T2 weighted (**a**), high b value 1500 DWI (**b**), ADC map (**c**) and dynamic contrast-enhanced (**d**) images show a decrease in size of the prostate gland with a volume of 24 cc (arrow). The peripheral zone demonstrates slightly more T2 hypointensity (**a**) (arrow) and slightly more restricted diffusion (**c**) (arrow). However, the transition zone has decreased in size and demonstrates less (**c**) restricted diffusion (arrowhead) and enhancement (arrow). The overall appearances are in keeping with resolving prostatitis

Repeat prostate MRI done four months following treatment initiation demonstrated resolving prostatitis (Fig. 4) and CT chest demonstrated resolution of para-aortic mass. Given his clinical improvement, invasive investigations such as cystoscopy or prostate biopsy were not pursued.

## Discussion and conclusions

IgG4-RD is an increasingly recognized fibroinflammatory disorder whose pathogenesis remains incompletely understood [1, 2]. B and T cells have been thought to be key players in disease progression, through production of IgG4-producing plasma cells and cytokines promoting regulatory and Th2 cell phenotypes, respectively [1, 16]. Patients with low disease burden in non critical organs such as submandibular glands can be observed, but those with symptomatic disease or involvement of critical organs such as aorta or biliary system require systemic therapy. As long as the disease is diagnosed early, before significant complications such as chronic pancreatic insufficiency or retroperitoneal fibrosis occur, the prognosis is quite good as the disease is generally responsive to treatment. Glucocorticoids are typically used as first-line therapy, with response rates of over 80%. The main toxicity is new or worsening diabetes in 30–40% of patients [1, 17]. Immunomodulatory therapy with the anti-CD20 antibody rituximab is effective in over 95% of patients, and other immunomodulators such as azathioprine, cyclophosphamide and mycophenolate mofetil may be useful as adjunctive therapies [1, 2, 18]. In rare cases of refractory disease, use of lymphoma or myeloma type chemotherapy has been reported [19].

IgG4-related prostatitis is a rare manifestation of IgG4-RD first described by Yoshimura et al. in 2006 [7]. The majority of cases involve males over the age of 55 and commonly present with voiding symptoms and dysuria [10]. Several case reports have also reported a possible association with prostate cancer [12, 13]. Diagnosis of IgG4-related prostatitis remains challenging as IgG4-RD clinically mimics infection, malignancy, and other inflammatory conditions [2]. SPEP and IgG subclasses are helpful investigations when IgG4-RD is suspected, but histology remains pivotal to diagnosis [19]. Furthermore, PSA is of limited utility as levels may be low, normal, or elevated, with low levels being attributed to acinar atrophy associated with IgG4-related prostatitis [10]. Response to therapy with steroids, rather than alpha-blocker therapy may also support the diagnosis of IgG4-related prostatitis [8, 9]. There have been no previous reports outlining duration of steroid or rituximab treatment required to achieve symptomatic improvement. ^18^F-Fluorodeoxyglucose (^18^F-FDG)-positron emission tomography (PET) has also been used to support diagnosis of IgG4-related prostatitis as IgG4 lesions are FDG-avid [14, 20].

Our case highlights the presence of polyclonal hypergammaglobulinemia as an early diagnostic indicator suggestive of IgG4-RD [21]. Over one year prior to developing voiding symptoms, in April 2020, beta-gamma bridging was noted on SPEP as well as elevated serum IgE and IgG levels. Indeed, patients with IgG4-RD may present with elevated serum IgE, IgG, and IgG4 levels [21], and production of IgG and IgG4 appears to be higher among Asian people than in Whites with IgG4-RD [19, 22].

Our case has several novel features that may be used to facilitate diagnosis of IgG4-related prostatitis non-invasively. Our patient’s medical history was notable for a one-year history of sinusitis. Individuals with IgG4-RD often have preceding histories of allergies, commonly including rhinitis, nasal polyps, asthma, as well as eosinophilia in up to 40% [2, 23]. Furthermore, our patient had a recent biopsy confirmed diagnosis of IgG4-RD in his submandibular mass. Reported cases of IgG4-related prostatitis in the literature rarely involve prostatitis as the initial organ affected, and presentation of prostatitis often occurs following involvement of other organs, such as the pancreas, salivary glands, and orbits [7, 9–11]. It is unclear whether this reflects underrecognition of IgG4-related prostatitis or delayed prostatic involvement in the natural history of IgG4-RD. Finally, responsiveness to steroids and resistance to alpha-blockers have been described as diagnostic features of IgG4-related prostatitis. Of note, our patient’s voiding symptoms resolved after 5 days of steroid therapy [8, 10]. Responsiveness to medications may be used as a diagnostic indicator of IgG4-RD prostatitis and potentially spare invasive definitive diagnostic procedures such as prostate biopsy. Further, recognition of IgG4-related prostatitis and initiation of steroid therapy may spare invasive treatments including transurethral resection of the prostate.

We report the youngest age of presentation with voiding symptoms attributable to IgG4-RD. Only one previous case by Vankadari et al. has reported a case of IgG4-related prostatitis in a 20-year-old male, however his presenting symptoms were recurring fever and abdominal pain, with no voiding symptoms [14]. Our case is also the first to report priapism in a patient with IgG4-RD. There appears to be an association between Peyronie’s disease, a fibroinflammatory condition of the corpus cavernosum, and serum IgG4 levels [24], but underlying mechanisms have not yet been elucidated. Given that priapism may rarely be associated with inflammation, further investigation into a possible association between priapism and IgG4-RD may be warranted.

Our case highlights the need to consider IgG4-related prostatitis as an etiology of urinary obstruction in young individuals and that resolution of symptoms after treatment with steroids may be diagnostic of IgG4-related prostatitis.

## Data Availability

Not applicable.

## Abbreviations

ACR: American College of Rheumatology
BPH: Benign prostatic hyperplasia
CT: Computed tomography
EULAR: European League Against Rheumatism
^18^F-FDG: ^18^F-fluorodeoxyglucose
IgG4-RD: Immunoglobulin G4-related disease
PET: Positron emission tomography
PSA: Prostate serum antigen
SPEP: Serum protein electrophoresis
